# Family History of Dementia in an Essential Tremor Cohort

**DOI:** 10.5334/tohm.1289

**Published:** 2026-10-22

**Authors:** Elan D. Louis, Stephanie Cosentino

**Affiliations:** 1 Department of Neurology, University of Texas Southwestern Medical Center, Dallas, Texas, USA; 2 Peter O’Donnell Jr. Brain Institute, University of Texas Southwestern Medical Center, Dallas, TX, USA; 3 Department of Epidemiology, Peter O’Donnell Jr. School of Public Health, University of Texas Southwestern Medical Center, Dallas, TX, USA; 4 Taub Institute for Research on Alzheimer’s Disease and the Aging Brain, Columbia University Irving Medical Center, New York, NY 10032, USA; 5 Gertrude H. Sergievsky Center, Columbia University Irving Medical Center, New York, NY 10032, USA; 6 Department of Neurology, Columbia University Irving Medical Center, New York, NY 10032, USA

**Keywords:** tremor, dementia, essential tremor, clinical, association, epidemiology, family history

Essential tremor (ET) is a neurodegenerative motor disease [1,2,3,4] whose defining clinical feature is kinetic tremor [5]. ET patients also have been shown to perform more poorly on cognitive testing than age-matched controls and furthermore, epidemiological studies have demonstrated that a diagnosis of ET significantly increases risk for dementia (relative risks = 1.64–1.89) [6,7]. In fact, longitudinal data indicate that conversion rate from normal cognition to dementia in older adults with ET is approximately three-times higher than in older adults without ET [8]. Hence, we are witnessing the emerging recognition of a new association – ET with dementia. The pathogenesis of this dementia is not clear, however initial studies suggest that there is a predisposition for ET patients to develop Alzheimer’s disease (AD) [9,10]. Although scientifically intriguing, given the very high prevalence of ET (2.2% of the entire US population – 7 million people) [11], the increased risk of dementia is troubling from both clinical and public health perspectives.

The association between ET and dementia has been studied using case-control and cohort studies, assessing the relative prevalence and/or incidence of dementia in ET versus control groups. Another pathway to address whether dementia is disease-associated in ET is to conduct a familial aggregation study. Familial aggregation studies ask whether *relatives of* ET cases have cognitive impairment rather than whether *ET cases themselves* have cognitive impairment, and whether this level of cognitive impairment exceeds that seen in relatives of controls. In the absence of a control group, the prevalence of dementia in ET relatives may be compared with the prevalence of dementia in relatives of historical controls.

Here, we report the results of such analyses. ET cases were enrolled in a prospective, longitudinal, nationwide study of older adults with ET (Clinical Pathological Study of Cognitive Impairment in Essential Tremor [COGNET]; National Institutes of Health Award #R01 NS086736), described in detail [8]. To date, cases reside in 43 US states, having responded to an advertisement to enroll in this cohort [8]. The Yale University, Columbia University, and University of Texas Southwestern Medical Center Institutional Review Boards approved the study protocol. All cases provided written, informed consent. During the in-home baseline evaluation, ET cases were asked “Do you have a family history of dementia?”

Data were available on 132 ET cases whose mean age was 76.5 years (range = 65–97 years). Of these, 55 (41.7%, 95% confidence interval = 33.2% to 50.6%) reported that a relative had dementia. Among these 132 were 43 (32.6%) who reported a first-degree relative with dementia and 15 (11.4%) who reported a second-degree relative with dementia. Among the 55 who reported relatives with dementia, 37 (67.3%) noted that they knew that dementia had been formally diagnosed by a physician. Among the 132 ET cases were 9 with Mild Cognitive Impairment and 3 with dementia; the proportion of relatives with dementia was reported as 7 (22.2%) and 1 (33.3%), respectively.

Published data on the proportion of controls who report a family history of dementia are as follows. In a worldwide study, 59,571 controls filled out an on-line survey; the proportion of controls reporting a first-degree relative with AD varied by age, but in 70–80 year olds ranged between 20% and 25% [12]. Similarly, in the Rotterdam study, 24.6% of 997 controls with a mean age of 68.4 years reported a family history of dementia [13]. A smaller study of 60 controls with a median age of 76 years reported that 13% had a family history of dementia [14]. All of these values are lower than those we report in our ET cohort.

Relatives of ET cases have rarely been compared with relatives of controls. In a prior study, investigators demonstrated similar mean Montreal Cognitive Assessment (MoCA) scores in 156 relatives of ET cases vs. 73 relatives of controls, but a higher prevalence of MoCA <26 in the former (p = 0.008), suggesting that there was some familial aggregation of lower cognitive test scores among relatives of ET cases [15]. These data are in sync with the data we report here on the substantial proportion of ET cases who report relatives with dementia.

Our analyses have a number of notable and important limitations. First, we were unable to compare our cases to controls, therefore relying on historical controls. Differences in study methodologies (e.g., the nature of the questions asked) make comparisons with historical controls susceptible to error. Second, our data focus on reported dementia rather than detailed neuropsychological test batteries in the relatives themselves. Third, the proportion of probands with positive family history can be a function of family size and family age; larger families and older families are more likely to have affected relatives.

These data demonstrate a high prevalence of reported dementia in ET families in our cohort, a value that is considerably higher than those reported in population-based studies of controls. Future familial aggregation studies, enrolling ET cases and controls, are needed to more fully address this issue. Greater understanding of the connections between ET and dementia is needed.

## References

[1] Grimaldi G, Manto M. Is essential tremor a Purkinjopathy? The role of the cerebellar cortex in its pathogenesis. Mov Disord. 2013;28:1759–1761. DOI: 10.1002/mds.25645 24114851

[2] Benito-Leon J. Essential tremor: a neurodegenerative disease? Tremor Other Hyperkinet Mov (N Y). 2014;4:252. DOI: 10.5334/tohm.227 PMC4107287 25120943

[3] Bonuccelli U. Essential tremor is a neurodegenerative disease. J Neural Transm. 2012;119:1383–1387. DOI: 10.1007/s00702-012-0878-8 23011236

[4] Lenka A, Pandey S. Essential Tremor: Five New Things. Neurol Clin Pract. 2022;12:183–186. DOI: 10.1212/CPJ.0000000000001145 PMC9208407 35747894

[5] Wagle Shukla A. Diagnosis and Treatment of Essential Tremor. Continuum (Minneap Minn). 2022;28:1333–1349. DOI: 10.1212/CON.0000000000001181 PMC12360682 36222768

[6] Bermejo-Pareja F, Louis ED, Benito-Leon J. Risk of incident dementia in essential tremor: A population-based study. Mov Disord. 2007;22:1573–1580. DOI: 10.1002/mds.21553 17516478

[7] Thawani SP, Schupf N, Louis ED. Essential tremor is associated with dementia: prospective population-based study in New York. Neurology. 2009;73:621–625. DOI: 10.1212/WNL.0b013e3181b389f1 PMC2731620 19704081

[8] Ghanem A, Berry DS, Burkes A, et al. Prevalence of and Annual Conversion Rates to Mild Cognitive Impairment and Dementia: Prospective, Longitudinal Study of an Essential Tremor Cohort. Ann Neurol. 2024;95:1193–1204. DOI: 10.1002/ana.26927 PMC11463725 38654628

[9] Wainman E, Berry DS, Hernandez NC, et al. Neuropathologic Changes in People With Essential Tremor With Dementia: A Postmortem Study. Neurology. 2025;105:e214310. DOI: 10.1212/WNL.0000000000214310 PMC12576839 41160795

[10] Saez-Calveras N, Vaquer-Alicea J, White CL 3rd, et al. Essential tremor with tau pathology features seeds indistinguishable in conformation from Alzheimer’s disease and primary age-related tauopathy. Acta Neuropathol. 2025;149:6. DOI: 10.1007/s00401-024-02843-6 PMC11711841 39775287

[11] Louis ED, Ottman R. How many people in the USA have essential tremor? Deriving a population estimate based on epidemiological data. Tremor Other Hyperkinet Mov (N Y). 2014;4:259. DOI: 10.5334/tohm.198 PMC4137360 25157323

[12] Talboom JS, Haberg A, De Both MD, et al. Family history of Alzheimer’s disease alters cognition and is modified by medical and genetic factors. Elife. 2019;8. DOI: 10.7554/eLife.46179 PMC6615857 31210642

[13] Slooter AJ, Cruts M, Kalmijn S, et al. Risk estimates of dementia by apolipoprotein E genotypes from a population-based incidence study: the Rotterdam Study. Arch Neurol. 1998;55:964–968. DOI: 10.1001/archneur.55.7.964 9678314

[14] Woodruff BK, Graff-Radford NR, Ferman TJ, et al. Family history of dementia is a risk factor for Lewy body disease. Neurology. 2006;66:1949–1950. DOI: 10.1212/01.wnl.0000219812.20616.b3 16801670

[15] Meyers JH, Hickman R, Cristal AD, Factor-Litvak P, Cosentino S, Louis ED. More unaffected first-degree relatives of essential tremor cases have mild cognitive deficits than age-matched controls. Parkinsonism Relat Disord. 2019;61:144–150. DOI: 10.1016/j.parkreldis.2018.10.030 PMC6488412 30404762

